# Steady Vortex Sheets in Presence of Surface Tension

**DOI:** 10.1007/s00021-026-01056-z

**Published:** 2026-09-07

**Authors:** Federico Murgante, Emeric Roulley, Stefano Scrobogna

**Affiliations:** 1 Università Statale di Milano, Via Saldini 50, 20133 Milano, Italy; 2 SISSA International School for Advanced Studies, Via Bonomea 265, 34136 Trieste, Italy; 3 Department of Mathematics and Geosciences, University of Trieste, via Valerio 12/1, 34127 Trieste, Italy

## Abstract

In this paper, we construct a new class of uniformly rotating vortex sheets arising as perturbations of the circular configuration within the Kelvin–Helmholtz model for two incompressible fluids of equal density, with surface tension effects taken into account. Despite the fundamental importance of the problem, few results are available on the long-time dynamics, due to the strong instability inherent in the Kelvin–Helmholtz mechanism. We carry out a detailed bifurcation analysis and construct families of nontrivial rotating solutions that bifurcate from the circular state when either the rotational speed or the surface tension coefficient is varied. The stationary branches obtained by varying the mean vorticity follow from the same analysis through the scaling symmetry of the equations.

## Introduction

Interfaces separating two inviscid fluids, whose evolution is described by the Kelvin-Helmholtz (KH) equations, often break into elaborate vortex patterns. More than one hundred and fifty years ago, Kelvin and Helmholtz predicted that an infinitesimally thin vortex sheet should tear itself apart under its own shear. Surface tension, even when small, stabilizes the high-frequency Kelvin–Helmholtz instability. This competition between vortical instability and capillary stabilization determines the fundamental character of the evolution equations for KH.

Mathematically, KH equations with surface tension enjoy short-time well-posedness, [1, 2]. However, the long-time or global dynamics remain largely unexplored: aside from trivial circles and lines, no closed curve solutions are known.

A major source of difficulty lies in the subtle interplay between surface tension effects and the underlying Kelvin–Helmholtz instability. Furthermore, when addressing the full nonlinear problem, one must deal with a quasilinear and nonlocal structure, which adds significant analytical complexity. The main goal of this work is to initiate a rigorous study of the long-time dynamics for KH by constructing a new class of uniformly rotating vortex sheets that persist *for all times* and remain arbitrarily close to the unit circle. Each branch bifurcates from the circular steady state when one of the physical parameters—the angular speed, the surface tension coefficient, or, for the stationary family, the mean vorticity—is varied. To our knowledge, this is the first rigorous demonstration that closed KH sheets with capillarity can survive indefinitely while rotating.

To this end, we consider a planar Euler system for two irrotational, incompressible fluids with the same density (normalized to 1), separated by an interface Γ(t) homeomorphic to a circle and parametrized by $z(t,\cdot ):\mathbb {T}\rightarrow \mathbb {R}^2.$ This interface divides the plane into two open components $\Omega ^{\pm }(t)$ with $\Omega ^{-}(t)$ bounded and $\Omega ^{+}(t)$ unbounded. The evolutionary system is thus composed of the following equations

1.1 $$\begin{aligned} {\left\{ \begin{array}{ll} u_t^{\pm }+u^{\pm }\cdot \nabla u^{\pm }+\nabla p^{\pm }=0, &  {\text {in }}\Omega ^{\pm }(t),\\ \big (z_t-u^{\pm }|_{\Gamma (t)}\big )\cdot z_{x}^\perp =0,&  {\text {at }}\Gamma (t),\\ {(p^--p^+)|_{\Gamma (t)}=-\sigma \mathcal {K}(z)}, &  {\text {at }}\Gamma (t),\\ u^{+}(t,\textbf{x}) \rightarrow 0, &  {\text {as }} |\textbf{x}|\rightarrow +\infty ,\\ \nabla \cdot u^{\pm }=0, &  {\text {in }}\Omega ^{\pm }(t),\\ \nabla ^{\perp }\cdot u^{\pm }=0, &  {\text {in }}\Omega ^{\pm }(t). \end{array}\right. } \end{aligned}$$

In the above set of equations, the quantities $u^{\pm },p^{\pm }$ are respectively the velocity field and pressure inside the domain $\Omega ^{\pm }.$ For a vector $z=(z_1,z_2)$ and a velocity field $u=(u_1,u_2)$, our conventions are

$$\begin{aligned} z^{\perp }\triangleq (-z_2,z_1),\qquad \nabla ^{\perp }\triangleq (-\partial _2,\partial _1),\qquad \operatorname {curl}u\triangleq \nabla ^{\perp }\cdot u=\partial _1u_2-\partial _2u_1. \end{aligned}$$

The parametrization $z(x)=e^{\textrm{i} x}$ is counterclockwise. With the curvature convention below one has $\mathcal {K}(e^{\textrm{i} x})=-1$. No arclength or constant-speed parametrization is assumed: every geometric formula retains the appropriate factors of $\left| z_x\right|$. The parameter $\sigma \geqslant 0$ is the surface tension coefficient and $\mathcal {K}(z)$ is the curvature defined by

$$\mathcal {K}(z)\triangleq -\frac{z_{x}^{\perp }\cdot z_{xx}}{|z_x|^3}\cdot $$

The last equation in (1.1) implies that the vorticity distribution $\boldsymbol{\omega }$ is localized on the curve Γ(t) at time *t*,  namely

1.2 $$\begin{aligned} \boldsymbol{\omega }(t,\textbf{x})=\omega (t,x)\delta \big (\textbf{x}-z(t,x)\big ),\qquad \textbf{x}\in \mathbb {R}^2,\quad x\in \mathbb {T}. \end{aligned}$$

Such a solution is called *vortex sheet*. The velocity fields are recovered through the so-called Biot-Savart law

1.3 $$\begin{aligned} u^{\pm }(t,\textbf{x})=\int _{\mathbb {T}}\frac{\big (\textbf{x}-z(t,y)\big )^{\perp }}{\big |\textbf{x}-z(t,y)\big |^2}\omega (t,y){\text {d}} y, \end{aligned}$$

where we used the following convention for the integral on the torus $\mathbb {T}$

$$\begin{aligned} \int _{\mathbb {T}}f(y){\text {d}} y\triangleq \frac{1}{2\pi }\int _{0}^{2\pi }f(y){\text {d}} y. \end{aligned}$$

The equations for the system (1.1) in the case in which the vorticity is a vortex sheet (i.e. (1.2)) are called the *Kelvin-Helmholtz system*. From (1.3) and standard suppression of the pressure via Leray projectors it is clear that the bulk quantities $u^\pm$ and $p^\pm$ can be expressed in terms of the interface quantities ω and *z*, thus recasting (1.1) as a system of contour dynamics equations (CDEs) on $\mathbb {T}$. The resulting system, derived in Appendix A.1, writes as

1.4 $$\begin{aligned} \left\{ \begin{aligned}&\left( z_t - {\text {BR}}(z)\omega \big . \right) \cdot z_x^\perp =0, \\&\omega _t = \left( \omega \ \frac{\left( z_t - {\text {BR}}(z)\omega \right) \cdot z_x}{\left| z_x\right| ^2} \right) _x - \sigma \left( \mathcal {K}\left( z \right) \right) _x. \end{aligned} \right. \end{aligned}$$

where the *Birkhoff-Rott integral operator* is defined by

$${\text {BR}}(z)\omega (t,x)\triangleq {\text {p.v.}}\int _{\mathbb {T}}\frac{\big (z(t,x)-z(t,y)\big )^{\perp }}{|z(t,x)-z(t,y)|^2}\omega (t,y){\text {d}} y.$$

Let us choose a parametrization in the form

1.5 $$\begin{aligned} z(t,x)=R(t,x)e^{\textrm{i} x},\qquad R(t,x)\triangleq \sqrt{1+2\eta (t,x)}. \end{aligned}$$

The choice of the parametrization (1.5), which is a graph on the unit circle, allows us to recast the system (1.4) in a more congenial form. The detailed computations are performed in Appendix A.2 and produce the system

1.6 $$\begin{aligned} \left\{ \begin{aligned}&\eta _t=-\frac{1}{2} \mathscr {H}(\eta )[\omega ],\\&\omega _t=-\left( \frac{\omega }{2}\mathscr {D}_0(\eta )[\omega ]\right) _x-\sigma \big (\mathscr {K}(\eta )\big )_x, \end{aligned} \right. \end{aligned}$$

where

1.7 $$\begin{aligned} \mathscr {H}(\eta )[\omega ]&\triangleq \eta _{x}\mathscr {D}_0(\eta )[\omega ]+\mathscr {H}_0(\eta )[\omega ],\end{aligned}$$

1.8 $$\begin{aligned} \mathscr {D}_0(\eta )[\omega ](x)&\triangleq {\text {p.v.}}\int _{\mathbb {T}}\frac{1-\sqrt{\frac{1+2\eta (y)}{1+2\eta (x)}}\cos (x-y)}{1+\eta (x)+\eta (y)-\sqrt{1+2\eta (x)}\sqrt{1+2\eta (y)}\cos (x-y)}\omega (y){\text {d}} y,\end{aligned}$$

1.9 $$\begin{aligned} \mathscr {H}_0(\eta )[\omega ](x)&\triangleq {\text {p.v.}}\int _{\mathbb {T}}\frac{\sqrt{1+2\eta (x)}\sqrt{1+2\eta (y)}\sin (x-y)}{1+\eta (x)+\eta (y)-\sqrt{1+2\eta (x)}\sqrt{1+2\eta (y)}\cos (x-y)}\omega (y){\text {d}} y,\end{aligned}$$

1.10 $$\begin{aligned} \mathscr {K}(\eta )&\triangleq \frac{\eta _{xx}-2\left( \frac{\eta _{x}}{R}\right) ^2}{\left( R^2+\left( \frac{\eta _{x}}{R}\right) ^2\right) ^{\frac{3}{2}}}-\left( R^2+\left( \frac{\eta _{x}}{R}\right) ^2\right) ^{-\frac{1}{2}}. \end{aligned}$$

Using the divergence free property of the flow, Stokes’ Theorem, the second equation in (1.1) and the first equation in (A.9), we get

$$\begin{aligned} 0=\int _{\Omega ^-(t)}\nabla \cdot u^-(t,\textbf{x})\,d\textbf{x}=\int _{0}^{2\pi }u^{-}\big (t,z(t,x)\big )\cdot z_x^\perp (t,x)\, d x =\int _{0}^{2\pi } z_t(t,x)\cdot z_x^\perp (t,x)\,dx =-\frac{d}{dt}\int _{0}^{2\pi }\eta (t,x)\,dx. \end{aligned}$$

Therefore, the space average of η is preserved and we impose

$$\begin{aligned} \int _{\mathbb {T}}\eta (x)dx=0. \end{aligned}$$

The second equation in (1.6) implies the conservation of the mean vorticity and in the sequel, we denote

$$\begin{aligned} \gamma \triangleq \int _{\mathbb {T}}\omega (x)dx. \end{aligned}$$

Let us introduce the velocity potential ψ through the relation

1.11 $$\begin{aligned} \omega =\gamma +\psi _x,\qquad {\text {i.e.}}\qquad \psi \triangleq \partial _{x}^{-1}(\omega -\gamma ). \end{aligned}$$

In the new variables $(\eta ,\psi )$, the system (1.6) becomes

1.12 $$\begin{aligned} \left\{ \begin{aligned}&\eta _t=-\frac{1}{2} \mathscr {H}(\eta )[\psi _x]-\frac{\gamma }{2} \mathscr {H}(\eta )[1],\\&\psi _t=-\frac{\psi _x+\gamma }{2}\mathscr {D}_0(\eta )[\psi _x+\gamma ]-\sigma \mathscr {K}(\eta ). \end{aligned} \right. \end{aligned}$$

Let us emphazise that to obtain the second equation in (1.12), we have integrated in space the second equation in (1.6). Therefore, the second equation is well-defined up to a time-dependent additive constant.

The system (1.12) can be reformulated in a Hamiltonian form, akin to the approach taken by [3] for perturbations of the flat interface. The Hamiltonian structure has been obtained in [4, Sec. 2.1] and is an important ingredient in the study of small divisors problems for PDEs to construct for instance quasi-periodic solutions [5–15] and a fundamental tool to study long time stability in presence of resonances [4, 16–20]. However, the Hamiltonian reformulation does not play an important role in our bifurcation analysis so we do not recall its expression. At last, we mention that the system (1.12) is invariant under the scaling

1.13 $$\begin{aligned} \psi (t,x)\mapsto \lambda \psi \left( \lambda t,x\right) ,\qquad \eta (t,x)\mapsto \eta \left( \lambda t,x\right) ,\qquad \gamma \mapsto \lambda \gamma ,\qquad \sigma \mapsto \lambda ^2\sigma . \end{aligned}$$

This is reminiscent of the scaling of the Euler equations.

### Main Results

We look for uniformly rotating vortex sheets, that is traveling wave solutions of the system (1.12), namely

$$\begin{aligned} (\eta ,\psi )(t,x)=(\check{\eta },\check{\psi })(x-ct),\qquad c\in \mathbb {R},\qquad \check{\eta },\check{\psi }\in L^2(\mathbb {T}). \end{aligned}$$

When c=0, the corresponding solutions are stationary. The equations (1.12) rewrite in this context (for simplicity of notations, we still denote $(\eta ,\psi )$ instead of $(\check{\eta },\check{\psi })$)

1.14 $$\begin{aligned} {\left\{ \begin{array}{ll} c\,\eta _x+\frac{1}{2} \mathscr {H}(\eta )[\psi _x]+\frac{\gamma }{2} \mathscr {H}(\eta )[1]=0,\\ c\,\psi _x+\frac{\psi _x+\gamma }{2}\mathscr {D}_0(\eta )[\psi _x+\gamma ]+\sigma \mathscr {K}(\eta )=0. \end{array}\right. } \end{aligned}$$

Given $\textbf{m}\in \mathbb {N}^*$, we say that a uniformly rotating vortex sheet is $\textbf{m}$-fold if the functions $\check{\eta }$ and $\check{\psi }$ are $\frac{2\pi }{\textbf{m}}$ periodic.

The couple $(\eta ,\psi )=(0,0)$ is a trivial solution of (1.12) for any values of the parameters $c,\sigma ,\gamma$. This solution corresponds to the family of circular stationary vortex sheets given by

$$\begin{aligned} z(x)=e^{\textrm{i} x},\qquad \omega \equiv \gamma ,\qquad u^{-}(\textbf{x})\equiv 0,\qquad u^{+}(\textbf{x})=\gamma \frac{\textbf{x}^\perp }{|\textbf{x}|^2}\cdot \end{aligned}$$

We refer the reader to Lemma 2.1 for more details. Our main result, stated below, gives the existence of global-in-time non-trivial solutions which are small amplitude perturbations of this stationary state.

#### Theorem 1.1

**(Local bifurcation of vortex sheets from the circular distribution)**

(i) Let σ>0, $\gamma \in \mathbb {R}$ and $\textbf{m}\in \mathbb {N}^*$. Assume that
  1.15 $$\begin{aligned} \frac{\gamma ^2-\sigma }{\sigma \textbf{m}^2}\not \in \mathbb {N}^* \end{aligned}$$
  supplemented by one of the following conditions
  - $$4\sigma (2-\sqrt{3})<\gamma ^2<4\sigma (2+\sqrt{3}).$$
  - $$\gamma ^2\in [0,\infty )\setminus \left[ 4\sigma (2-\sqrt{3}),4\sigma (2+\sqrt{3})\right] $$ and $$\textbf{m}\in \mathbb {R}\setminus [m_-(\sigma ,\gamma ),m_+(\sigma ,\gamma )],$$ with
    $$\begin{aligned} m_{\pm }(\sigma ,\gamma )=\frac{\gamma ^2}{4\sigma }\pm \frac{1}{4\sigma }\sqrt{(\gamma ^2-8\sigma )^2-48\sigma ^2}\cdot \end{aligned}$$
  Then, as the angular speed is varied, two branches of $\textbf{m}$-fold uniformly rotating vortex sheets bifurcate from the circular distribution at
  $$\begin{aligned} c_{\textbf{m}}^{\pm }(\sigma ,\gamma )=-\frac{\gamma }{2}\pm \frac{1}{2}\sqrt{2\sigma \textbf{m}-\gamma ^2+\frac{2(\gamma ^2-\sigma )}{\textbf{m}}}\cdot \end{aligned}$$
(ii) Let $(c,\gamma )\in \mathbb {R}^2\setminus \{(0,0)\}.$ Fix $\textbf{m}\in \mathbb {N}\setminus \{0,1\}$ such that
  $$\begin{aligned} \textbf{m}>\frac{2\gamma ^2}{\left( 2c+\gamma \right) ^2+\gamma ^2}\cdot \end{aligned}$$
  Assume in addition that
  1.16 $$\begin{aligned} {\frac{(2\textbf{m}-1)\gamma ^2-(2c+\gamma )^2}{\textbf{m}\big (\textbf{m}(2c+\gamma )^2+(\textbf{m}-2)\gamma ^2\big )}\not \in \mathbb {N}^*.} \end{aligned}$$
  Then, as the surface tension coefficient is varied, one branch of $\textbf{m}$-fold uniformly rotating vortex sheets with speed *c* bifurcates from the circular distribution at
  $$\begin{aligned} \sigma _{\textbf{m}}(c,\gamma )=\frac{\textbf{m}\left( 2c+\gamma \right) ^2+(\textbf{m}-2)\gamma ^2}{2(\textbf{m}^2-1)}\cdot \end{aligned}$$

#### Corollary 1.1

Let σ>0 and $\textbf{m}\in \mathbb {N}\setminus \{0,1\}.$ Then there exist two branches of $\textbf{m}$-fold stationary vortex sheets bifurcating from the circular distribution at

$$\begin{aligned} \gamma _{\textbf{m}}^{\pm }(\sigma )=\pm \sqrt{\sigma (\textbf{m}+1)}\cdot \end{aligned}$$

#### Proof

Fix a sign $\kappa \in \{-,+\}$ and set $\gamma _0\triangleq \gamma _{\textbf{m}}^{\kappa }(\sigma )$. For c=0, the quotient in (1.16) equals $1/\textbf{m}$, and hence the second part of Theorem 1.1 applies at

$$\begin{aligned} \sigma _{\textbf{m}}(0,\gamma _0)=\frac{\gamma _0^2}{\textbf{m}+1}=\sigma . \end{aligned}$$

It produces a stationary branch $\texttt{s}\mapsto (\sigma (\texttt{s}),\eta (\texttt{s}),\psi (\texttt{s}))$ with $\sigma (0)=\sigma$ and fixed mean vorticity $\gamma _0$. Applying the scaling (1.13) with $\lambda (\texttt{s})\triangleq \sqrt{\sigma /\sigma (\texttt{s})}$ gives a stationary branch at fixed surface tension σ and mean vorticity $\gamma (\texttt{s})=\lambda (\texttt{s})\gamma _0$. Repeating the argument for both signs proves the claim. □

#### Remark 1.1

Let us make some remarks.

1. The condition (1.15) is satisfied in particular for
  $$\begin{aligned} \frac{\gamma ^2}{\sigma }\in \mathbb {R}{\setminus }\mathbb {Q}. \end{aligned}$$
2. Up to the normalization adopted here, the dimensionless ratio $\gamma ^2/\sigma$ is the Weber number. It measures the competition between inertial effects generated by the sheet strength and capillarity and is a natural parameter in the initial-value analysis of (1.12); see, for instance, [1, 4, 21].
3. The corrected condition (1.16) is compatible with c=0: in that case its left-hand side equals $1/\textbf{m}$. Moreover, $\sigma _{\textbf{m}}(0,\gamma )=\gamma ^2/(\textbf{m}+1)$, which yields Corollary 1.1 through the scaling (1.13). This is why Theorem 1.1 is formulated in terms of only two primary bifurcation scenarios.
4. The scaling (1.13) shows that the previous bifurcations are not independent. In our analysis this is reflected by the algebraic link between the parameters $(c,\sigma ,\gamma )$ (see (2.29))
  $$\begin{aligned} 4\textbf{m}\left( c+\tfrac{\gamma }{2}\right) ^2=2\sigma \textbf{m}^2-\gamma ^2\textbf{m}+2(\gamma ^2-\sigma ). \end{aligned}$$
5. For the family of solutions in Corollary 1.1, an explicit computation gives that at the first order with respect to the bifurcation parameter (i.e. the amplitude of the geometrical deformation), the velocity field $u^-$ vanishes. Therefore, it seems that our solutions coincide with the explicit ones found in [22] with a different approach–which might be confirmed by invoking the uniqueness of the constructed branch.

The Theorem 1.1 is obtained by means of local bifurcation theory, more precisely by applying Crandall-Rabinowitz Theorem A.1. For this aim, we seek solutions to (1.14) as zeros of a nonlinear functional $\mathcal {F}$ (defined by (2.1)-(2.2)):

$$\begin{aligned} \mathcal {F}(c,\sigma ,\gamma ,\eta ,\psi )=0. \end{aligned}$$

We prove in Lemma 2.1 that $(\eta ,\psi )=(0,0)$ is indeed a solution. Then, in Proposition 2.1, we study the regularity of $\mathcal {F}$ and we give in Proposition 2.2 the expression of its differential $\mathcal {L}_{c,\sigma ,\gamma }$ at the trivial solution $(\eta ,\psi )=(0,0).$ The two primary bifurcation analyses, with respect to *c* and σ, are carried out in Subsections 2.2 and 2.3. For completeness, Subsection 2.4 gives a direct Crandall–Rabinowitz verification of the stationary consequence in Corollary 1.1. Let us emphasize that the restrictions (1.15) and (1.16) are made for avoiding spectral collisions and get a one dimensional kernel for $\mathcal {L}_{c,\sigma ,\gamma }.$ The transversality conditions are obtained via a duality argument as developed in [23–26].

### Known Results

The study of the motion of two immiscible fluids separated by a sharp interface has been a subject of study since the mid-19th century, when von Helmholtz derived the evolution equations for such phenomenon in 1858 in [27]. When there is no surface tension and no gravity the problem is generally ill-posed in Sobolev spaces, even at linear level (cf. [28, Section 9.3]), this phenomenon, known as *Kelvin-Helmholtz instability*, lead to the formation of vortices or waves at the interface or within the shear layer. It is named after Lord Kelvin (William Thomson) and Hermann von Helmholtz, who independently investigated the instability in the late 19th century.

When the fluid interface is a perturbation of the flat rest state and there are no surface tension effects (σ=0) stability in the analytic framework was proved in [29–31]. Adding the effects of the surface tension to (1.1) (σ>0) induces stabilizing effects and the resulting equations are well-posed, linearly [21], and nonlinearly [1, 2, 32–35], even in presence of two fluids with different densities. A more comprehensive stability criterion has been investigated by D. Lannes in [36] in the context of two fluids with nonzero density, when gravitational effects are taken in consideration. We highlight that when we have a shear flow the presence of the surface tension is crucial in order to avoid the growth of high-modes, that is characteristically induced by the Kelvin-Helmholtz instability, otherwise the equations are ill-posed [37–39], in sharp contrast with what happens in the context of the Water-Waves equations. We mention as well the foundational work of Delort [40] which proved the existence of global weak solutions for measured-valued vorticities that include the vortex sheet problem.

The stability results discussed are constrained by an existence time proportional to the capillarity coefficient σ, leaving the long-term stability of (1.12) largely unexplored. When σ=0, circles and lines are non-trivial steady solutions, as are segments of length 2*a* with strength $\omega (x) = \Omega \sqrt{a^2-x^2}$, rotating uniformly at angular velocity Ω. This category also includes the family of solutions identified by B. Protas and T. Sakajo [41] and the solutions [42, 43] of Cao-Qin-Zou. When Γ(t) is a closed curve and σ=0, recent findings [44] have demonstrated that steady solutions bifurcate from degenerate eigenvalues. In [45], the same authors proved rigidity results, namely the non-existence of non-trivial solutions for a certain range of angular speed. Over the past decades, bifurcation techniques have been successfully employed to construct traveling periodic solutions near the flat equilibrium configuration [46, 47], as well as in the study of the three-dimensional liquid drop model with capillarity [48]. They have also been applied to the vortex patch setting, where a broad class of steady solutions of the CDEs has been discovered, beginning with Burbea’s seminal work [49] and extended in [50–60]. The previous list is non-exhaustive since restricted to the Eulerian model. Let us mention the recent generalization to a large class of models [61]. This manuscript adds to this literature by presenting an existence result for the Kelvin-Helmholtz equations near a circular vortex with surface tension effects. The combined contributions of our study and [44] establish, for the first time, the presence of closed curves as global solutions to Kelvin-Helmholtz equations.

## Proof of the Results

This section is devoted to the proof of Theorem 1.1. We first introduce the functional of interest and give the expression of its differential at the circular distribution. Then, we study the bifurcations with respect to the various parameters of the problem.

### Functional of Interest and Its Linear Operator

Since the second equation in (1.12) is defined modulo spatial constants, we identify an even function modulo constants with its zero-mean representative. More precisely, let

$$\begin{aligned} \Pi _0^\perp f\triangleq f-\int _{\mathbb {T}}f(x){\text {d}}x. \end{aligned}$$

Let us introduce the functional

$$\begin{aligned} \mathcal {F}=(\mathcal {F}_1,\mathcal {F}_2), \end{aligned}$$

where

2.1 $$\begin{aligned} \mathcal {F}_1(c,\sigma ,\gamma ,\eta ,\psi )&\triangleq c\,\eta _x+\frac{1}{2} \mathscr {H}(\eta )[\psi _x]+\frac{\gamma }{2} \mathscr {H}(\eta )[1],\end{aligned}$$

2.2 $$\begin{aligned} \mathcal {F}_2(c,\sigma ,\gamma ,\eta ,\psi )&\triangleq {\Pi _0^\perp \left( c\,\psi _x+\frac{\psi _x+\gamma }{2}\mathscr {D}_0(\eta )[\psi _x+\gamma ]+\sigma \mathscr {K}(\eta )\right) .} \end{aligned}$$

Then, clearly solutions of (1.14) are zeros of the functional $\mathcal {F}$ and we have the following result stating that the circular distribution is indeed a trivial solution for any values of the parameters.

#### Lemma 2.1

The state $(\eta ,\psi )=(0,0)$ is a trivial solution. More precisely,

$$\begin{aligned} \forall (c,\sigma ,\gamma )\in \mathbb {R}\times (0,\infty )\times \mathbb {R},\quad \mathcal {F}(c,\sigma ,\gamma ,0,0)=0. \end{aligned}$$

This corresponds to

2.3 $$\begin{aligned} \omega \equiv \gamma ,\qquad \Omega ^{-}(t)=D(0,1)\qquad {\text {and}}\qquad {\left\{ \begin{array}{ll} u^{-}(\textbf{x})=0, &  {\text {if }}\textbf{x}\in D(0,1),\\ u^{+}(\textbf{x})=\gamma \frac{\textbf{x}^\perp }{|\textbf{x}|^2}, &  {\text {if }}\textbf{x}\in \mathbb {R}^2\setminus \overline{D(0,1)}. \end{array}\right. }\end{aligned}$$

#### Proof

From (1.8) and (1.10), one readily gets

2.4 $$\begin{aligned} \mathscr {D}_0(0)[f](x)=\int _{\mathbb {T}}f(y){\text {d}} y,\qquad {\mathscr {K}(0)=-1.} \end{aligned}$$

Then, using the classical relations

2.5 $$\begin{aligned} \sin (u)=2\sin \left( \tfrac{u}{2}\right) \cos \left( \tfrac{u}{2}\right) ,\qquad 1-\cos (u)=2\sin ^2\left( \tfrac{u}{2}\right) , \end{aligned}$$

we obtain from (1.7) and (1.9)

2.6 $$\begin{aligned} \mathscr {H}(0)[f](x)=\mathscr {H}_0(0)[f](x)={\text {p.v.}}\int _{\mathbb {T}}\frac{\sin (x-y)}{1-\cos (x-y)}f(y){\text {d}} y={\text {p.v.}}\int _{\mathbb {T}}\cot \left( \frac{x-y}{2}\right) f(y){\text {d}} y=\mathcal {H}[f](x), \end{aligned}$$

where $\mathcal {H}$ denotes the 2π-periodic Hilbert transform. Inserting the foregoing calculations into (2.1)–(2.2) gives

$$\begin{aligned} \mathcal {F}_1(c,\sigma ,\gamma ,0,0)=\frac{\gamma }{2}\mathscr {H}(0)[1]=\frac{\gamma }{2}\mathcal {H}[1]=0 \end{aligned}$$

and

$$\begin{aligned} \mathcal {F}_2(c,\sigma ,\gamma ,0,0)=\Pi _0^\perp \left( \frac{\gamma ^2}{2}\mathscr {D}_0(0)[1]+\sigma \mathscr {K}(0)\right) =\Pi _0^\perp \left( \frac{\gamma ^2}{2}-\sigma \right) =0. \end{aligned}$$

Now let us turn to the fluid description. We employ the usual identification $\mathbb {R}^2\simeq \mathbb {C}$. For $(\eta ,\psi )=0$, (1.5) and (1.11) imply that

$$\begin{aligned} \omega \equiv \gamma \qquad {\text {and}}\qquad z(x)=e^{\textrm{i} x}. \end{aligned}$$

Note that

2.7 $$\begin{aligned} \frac{z_x(x)}{\textrm{i} z(x)}\equiv 1. \end{aligned}$$

We compute the complex conjugate of the velocity field for $\textbf{x}\in \mathbb {C}\setminus \partial D(0,1)$ which, in view of (1.3), is given by the following holomorphic function

$$\begin{aligned} \overline{u^{\pm }(\textbf{x})}&=\frac{\gamma }{2\pi \textrm{i} }\int _{0}^{2\pi }\frac{1}{\textbf{x}-z(x)}dx{\mathop {=}\limits ^{(2.7)}}\frac{\gamma }{\textrm{i} }\frac{1}{2\pi \textrm{i} }\int _{0}^{2\pi }\frac{z_x(x)}{(\textbf{x}-z(x))z(x)}dx=\frac{\gamma }{\textrm{i} }\frac{1}{2\pi \textrm{i} }\int _{\partial D(0,1)}\frac{dz}{(\textbf{x}-z)z}\\&={\left\{ \begin{array}{ll} 0,& \textbf{x}\in D(0,1),\\ \frac{\gamma }{\textrm{i} \,\textbf{x}}, &  \textbf{x}\not \in D(0,1), \end{array}\right. } \end{aligned}$$

where, to obtain the last equality, we used the residue Theorem. Taking the complex conjugate, we get

$$\begin{aligned} u^{-}(\textbf{x})=0,\qquad u^{+}(\textbf{x})=\gamma \frac{\textrm{i} }{\overline{\textbf{x}}}=\gamma \frac{\textrm{i} \textbf{x}}{|\textbf{x}|^2}=\gamma \frac{\textbf{x}^\perp }{|\textbf{x}|^2}\cdot \end{aligned}$$

This ends the proof of Lemma 2.1. □

We consider the following $\textbf{m}$-fold ($\textbf{m}\in \mathbb {N}^*$) Sobolev spaces with regularity index s⩾0,

$$\begin{aligned} H_{{\text {\tiny {even}}},\textbf{m}}^{s}&\triangleq \left\{ f(x)=\sum _{n=1}^{\infty }a_n\cos (n\textbf{m}x),\quad \sum _{n=1}^{\infty }\langle n\rangle ^{2s}|a_n|^2<\infty ,\quad a_n\in \mathbb {R}\right\} ,\\ H_{{\text {\tiny {odd}}},\textbf{m}}^{s}&\triangleq \left\{ f(x)=\sum _{n=1}^{\infty }b_n\sin (n\textbf{m}x),\quad \sum _{n=1}^{\infty }\langle n\rangle ^{2s}|b_n|^2<\infty ,\quad b_n\in \mathbb {R}\right\} , \end{aligned}$$

where we have used the classical notation $\langle n\rangle \triangleq \max (1,n).$ Then we set

$$\begin{aligned} X_{\textbf{m}}^s\triangleq H_{{\text {\tiny {even}}},\textbf{m}}^{s+\frac{1}{4}}\times H_{{\text {\tiny {odd}}},\textbf{m}}^{s-\frac{1}{4}},\qquad Y_{\textbf{m}}^{s}\triangleq H_{{\text {\tiny {odd}}},\textbf{m}}^{s-\frac{5}{4}}\times H_{{\text {\tiny {even}}},\textbf{m}}^{s-\frac{7}{4}} \end{aligned}$$

and for r>0,

$$\mathcal {B}_{\textbf{m}}^{s}(r)\triangleq \Big \{(\eta ,\psi )\in X_{\textbf{m}}^{s}\quad {\text {s.t.}}\quad \Vert \eta \Vert _{H^{s+\frac{1}{4}}}+\Vert \psi \Vert _{H^{s-\frac{1}{4}}}<r\Big \}.$$

In the next proposition, we state the regularity of the functional $\mathcal {F}$ with respect to these function spaces.

#### Proposition 2.1

Let $\textbf{m}\in \mathbb {N}^*$. There exists $s_0>0$ such that, for every $s\geqslant s_0$, there exists $r=r\left( s \right) >0$ for which the functional

$$\begin{aligned} \mathcal {F}:\mathbb {R}\times (0,\infty )\times \mathbb {R}\times \mathcal {B}_{\textbf{m}}^s(r)\longrightarrow Y_{\textbf{m}}^{s}, \end{aligned}$$

defined in (2.1)–(2.2), is well defined and of class $C^2$.

#### Proof

Let η be even and ψ odd. Then $\eta _x$ is odd and $\psi _x$ is even. In (1.8)-(1.9), perform simultaneously the changes (x,y)↦(-x,-y). Since $\eta (-x)=\eta (x)$, the denominator is unchanged, the numerator of $\mathscr {D}_0$ is unchanged, and the sine in the numerator of $\mathscr {H}_0$ changes sign. Hence, for every even function *f*,

2.8 $$\begin{aligned} \mathscr {D}_0(\eta )[f]\ {\text {is even}}, \qquad \mathscr {H}_0(\eta )[f]\ {\text {is odd}}. \end{aligned}$$

The identity (1.7) then shows that $\mathscr {H}(\eta )[f]$ is odd. The same calculation after the translation $(x,y)\mapsto (x+2\pi /\textbf{m},y+2\pi /\textbf{m})$ proves preservation of the $\textbf{m}$-fold symmetry. Finally, $\Pi _0^\perp$ preserves evenness and $\textbf{m}$-fold symmetry.

We next prove the Sobolev regularity. Put

$$\begin{aligned} \rho \triangleq s-\frac{5}{4}, \qquad a\triangleq 1+2\eta , \qquad q\triangleq \frac{\eta _x}{a}, \qquad \Delta _z\eta (x)\triangleq \frac{\eta (x)-\eta (x-z)}{2\sin (z/2)}, \qquad q_z\triangleq \frac{\Delta _z\eta }{a}. \end{aligned}$$

We take $s_0$ sufficiently large that ρ>1/2. For *r* sufficiently small, *a* is uniformly positive and $q_z$ takes values in a fixed interval $[-\delta ,\delta ]$, with δ<1/4, uniformly in *z*.

Set $t_z\triangleq \sin (z/2)$ and, for $|X|\leqslant \delta$,

$$\begin{aligned} S_z(X)\triangleq \sqrt{1-4Xt_z}, \qquad A_z(X)\triangleq S_z(X)+\frac{4X^2}{\left( 1+S_z(X) \right) ^2}. \end{aligned}$$

Using y=x-z and the identity

$$\begin{aligned} 1-2Xt_z-S_z(X)\cos z=2t_z^2A_z(X), \end{aligned}$$

the kernels in (1.8)–(1.9) take the form

2.9 $$\begin{aligned} \mathscr {H}_0(\eta )[f](x)&={\text {p.v.}}\int _{\mathbb {T}} \textsf{K}_z\left( q_z(x) \right) \cot (z/2)f(x-z){\text {d}}z, \end{aligned}$$

2.10 $$\begin{aligned} \mathscr {D}_0(\eta )[f](x)&={\text {p.v.}}\int _{\mathbb {T}} \frac{\textsf{G}_z\left( q_z(x) \right) }{a(x)t_z} f(x-z){\text {d}}z, \end{aligned}$$

where

$$\begin{aligned} \textsf{K}_z(X)\triangleq \frac{S_z(X)}{A_z(X)}, \qquad \textsf{G}_z(X)\triangleq \frac{\frac{2X}{1+S_z(X)}+S_z(X)t_z}{A_z(X)}. \end{aligned}$$

These functions are analytic in (*z*, *X*) on $[-\pi ,\pi ]\times [-\delta ,\delta ]$, and

$$\begin{aligned} \textsf{K}_0(X)=\textsf{k}(X)\triangleq \frac{1}{1+X^2}, \qquad \textsf{G}_0(X)=\textsf{g}(X)\triangleq \frac{X}{1+X^2}. \end{aligned}$$

Consequently,

2.11 $$\begin{aligned} \mathscr {H}_0(\eta )[f]&=\textsf{k}(q)\mathcal {H}[f] +\int _{\mathbb {T}} \textsf{R}_{\mathscr {H}}(\eta ;\cdot ,z) f(\cdot -z){\text {d}}z, \end{aligned}$$

2.12 $$\begin{aligned} \mathscr {D}_0(\eta )[f]&=\frac{\textsf{g}(q)}{a}\mathcal {H}[f] +\int _{\mathbb {T}} \textsf{R}_{\mathscr {D}}(\eta ;\cdot ,z) f(\cdot -z){\text {d}}z, \end{aligned}$$

with

$$\begin{aligned} \textsf{R}_{\mathscr {H}}(\eta ;x,z)&\triangleq \cot (z/2) \left[ \textsf{K}_z\left( q_z(x) \right) -\textsf{k}\left( q(x) \right) \right] ,\\ \textsf{R}_{\mathscr {D}}(\eta ;x,z)&\triangleq \frac{1}{a(x)t_z} \left[ \textsf{G}_z\left( q_z(x) \right) -\cos (z/2)\textsf{g}\left( q(x) \right) \right] . \end{aligned}$$

In particular, $$\mathcal {H}[\psi _x]=|D|\psi $$.

We now estimate the remainders at the regularity available in $X_{\textbf{m}}^s$. The Fourier multipliers defining $\Delta _z$ satisfy

2.13 $$\begin{aligned} \sup _{z\in \mathbb {T}} \Vert \Delta _z h\Vert _{H^{\rho +1/2}} + \sup _{0<|z|\leqslant \pi } |z|^{-1/2} \Vert \Delta _z h-h_x\Vert _{H^\rho } \leqslant C_s\Vert h\Vert _{H^{\rho +3/2}}. \end{aligned}$$

Indeed, their symbols are bounded respectively by *C*|*n*| and $C|z|^{1/2}|n|^{3/2}$. Since ρ>1/2, Moser estimates and differentiation of $q_z=\Delta _z\eta /a$ give, for j=0,1,2,

2.14 $$\begin{aligned}&\sup _{z\in \mathbb {T}} \left\| \partial _\eta ^j q_z[h_1,\ldots ,h_j] \right\| _{H^{\rho +1/2}} + \sup _{0<|z|\leqslant \pi } |z|^{-1/2} \left\| \partial _\eta ^j \left( q_z-q \right) [h_1,\ldots ,h_j] \right\| _{H^\rho } \nonumber \\&\hspace{4cm} \leqslant C_s\left( 1+\Vert \eta \Vert _{H^{\rho +3/2}} \right) \prod _{\ell =1}^j \Vert h_\ell \Vert _{H^{\rho +3/2}}. \end{aligned}$$

Moreover, analyticity gives, for $0\leqslant \ell \leqslant 3$,

$$\begin{aligned} \sup _{|X|\leqslant \delta } \left( \left| \partial _X^\ell \left( \textsf{K}_z-\textsf{k} \right) (X) \right| + \left| \partial _X^\ell \left( \textsf{G}_z-\textsf{g} \right) (X) \right| \right) \leqslant C_\ell |z|. \end{aligned}$$

To estimate $\textsf{R}_{\mathscr {H}}$, we split

$$\begin{aligned} \textsf{K}_z(q_z)-\textsf{k}(q) = \left[ \textsf{K}_z(q_z)-\textsf{k}(q_z) \right] + \left[ \textsf{k}(q_z)-\textsf{k}(q) \right] . \end{aligned}$$

For $\textsf{R}_{\mathscr {D}}$ we use the analogous decomposition

$$\begin{aligned} \textsf{G}_z(q_z)-\cos (z/2)\textsf{g}(q) = \left[ \textsf{G}_z(q_z)-\textsf{g}(q_z) \right] + \left[ \textsf{g}(q_z)-\textsf{g}(q) \right] + \left[ 1-\cos (z/2) \right] \textsf{g}(q). \end{aligned}$$

Combining these identities with (2.14), $|t_z|\gtrsim |z|$ near the origin, and standard composition estimates yields, for j=0,1,2,

2.15 $$\begin{aligned}&\int _{\mathbb {T}} \left( \left\| \partial _\eta ^j \textsf{R}_{\mathscr {H}} (\eta ;\cdot ,z) [h_1,\ldots ,h_j] \right\| _{H^\rho } + \left\| \partial _\eta ^j \textsf{R}_{\mathscr {D}} (\eta ;\cdot ,z) [h_1,\ldots ,h_j] \right\| _{H^\rho } \right) {\text {d}}z \nonumber \\&\hspace{4cm} \leqslant C_s\left( 1+\Vert \eta \Vert _{H^{\rho +3/2}} \right) \prod _{\ell =1}^j \Vert h_\ell \Vert _{H^{\rho +3/2}}. \end{aligned}$$

The only singular contribution in this estimate is $O\left( |z|^{-1/2} \right)$, hence it is integrable. Since translations are isometries on $H^\rho$ and $H^\rho$ is an algebra, Minkowski’s inequality, (2.11), (2.12), and (2.15) imply

2.16 $$\begin{aligned} (\eta ,f) \longmapsto \left( \mathscr {H}_0(\eta )[f], \mathscr {D}_0(\eta )[f] \right) \in C^2\left( B_{H^{s+1/4}}(0,r)\times H^\rho ; H^\rho \times H^\rho \right) . \end{aligned}$$

This applies to $f=\psi _x$ and to f=1, and linearity covers $f=\psi _x+\gamma$. The term $\eta _x\mathscr {D}_0(\eta )[f]$ in (1.7) is controlled by the same product estimates.

It remains to treat the curvature. Write

$$\begin{aligned} Q(\eta )\triangleq a+\frac{\eta _x^2}{a}, \qquad N(\eta )\triangleq \eta _{xx}-2\frac{\eta _x^2}{a}, \qquad \mathscr {K}(\eta ) = N(\eta )Q(\eta )^{-3/2}-Q(\eta )^{-1/2}. \end{aligned}$$

For a variation *h*,

$$\begin{aligned} dQ(\eta )[h]&= 2h+2\frac{\eta _xh_x}{a} -2\frac{\eta _x^2h}{a^2},\\ dN(\eta )[h]&= h_{xx}-4\frac{\eta _xh_x}{a} +4\frac{\eta _x^2h}{a^2},\\ d\mathscr {K}(\eta )[h]&= Q^{-3/2}dN(\eta )[h] -\frac{3}{2}NQ^{-5/2}dQ(\eta )[h] +\frac{1}{2}Q^{-3/2}dQ(\eta )[h]. \end{aligned}$$

Since *Q* is uniformly positive on a sufficiently small ball, a second differentiation and the Moser estimates give

2.17 $$\begin{aligned} \eta \longmapsto \mathscr {K}(\eta ) \in C^2\left( B_{H^{s+1/4}}(0,r); H^{s-7/4} \right) . \end{aligned}$$

Combining (2.8), (2.16), (2.17), the boundedness of $\Pi _0^\perp$, and standard product estimates proves that $\mathcal {F}$ maps into $Y_{\textbf{m}}^s$ and is $C^2$ in $(\eta ,\psi )$. Its dependence on $(c,\sigma ,\gamma )$ is polynomial, so the full map is $C^2$ as claimed. □

Our next goal is to linearize the system (1.14) at the trivial solution $(\eta ,\psi )=(0,0).$ The corresponding operator has a good Fourier multiplier structure and enjoys the Fredholm property with respect to the function spaces introduced above. More precisely, we have the following proposition.

#### Proposition 2.2

Let $(c,\sigma ,\gamma )\in \mathbb {R}\times (0,\infty )\times \mathbb {R}.$ We denote

$$\mathcal {L}_{c,\sigma ,\gamma }\triangleq d_{\eta ,\psi }\mathcal {F}(c,\sigma ,\gamma ,0,0).$$

(i) The operator $\mathcal {L}_{c,\sigma ,\gamma }$ writes
  2.18 $$\begin{aligned} \mathcal {L}_{c,\sigma ,\gamma }=\begin{pmatrix} \left( c+\tfrac{\gamma }{2}\right) \partial _{x} &  \tfrac{1}{2}|D|\\ \sigma -\gamma ^2+\tfrac{\gamma ^2}{2}|D|-\sigma |D|^2&  \left( c+\tfrac{\gamma }{2}\right) \partial _{x} \end{pmatrix}. \end{aligned}$$
  In particular, it is a Fourier multiplier. Its action on $(\hat{\eta },\hat{\psi }),$ admitting the Fourier expansions
  $$\begin{aligned} \hat{\eta }(x)=\sum _{n=1}^{\infty }a_n\cos (n\textbf{m}x),\qquad \hat{\psi }(x)=\sum _{n=1}^{\infty }b_n\sin (n\textbf{m}x),\qquad a_n,b_n\in \mathbb {R}, \end{aligned}$$
  is given by
  $$\mathcal {L}_{c,\sigma ,\gamma }\begin{pmatrix} \hat{\eta }\\ \hat{\psi } \end{pmatrix}(x)=\sum _{n=1}^{\infty }\begin{pmatrix} \sin (n\textbf{m}x) &  0\\ 0 &  \cos (n\textbf{m}x) \end{pmatrix}M_{n\textbf{m}}(c,\sigma ,\gamma )\begin{pmatrix} a_n\\ b_n \end{pmatrix},$$
  with
  2.19 $$\begin{aligned} M_n(c,\sigma ,\gamma )\triangleq \begin{pmatrix} -\left( c+\frac{\gamma }{2}\right) n &  \frac{n}{2}\\ \sigma -\gamma ^2+\frac{\gamma ^2}{2}n-\sigma n^2 &  \left( c+\frac{\gamma }{2}\right) n \end{pmatrix}. \end{aligned}$$
(ii) The operator $\mathcal {L}_{c,\sigma ,\gamma }:X_{\textbf{m}}^{s}\longrightarrow Y_{\textbf{m}}^{s}$ is Fredholm with zero index.

#### Proof

(*i*) First observe that from the expression (1.10), one readily gets

2.20 $$\begin{aligned} d_{\eta }\mathscr {K}(0)[\hat{\eta }]=\hat{\eta }+\hat{\eta }_{xx}={({\text {Id}}-|D|^2)\hat{\eta }.} \end{aligned}$$

Then, differentiating in (1.9), we infer

2.21 $$\begin{aligned} d_{\eta }\mathscr {H}_0(0)[\hat{\eta }][f](x)=0. \end{aligned}$$

Now, differentiating in (1.8) and using one more time (2.5) yields

2.22 $$\begin{aligned} \begin{aligned} d_{\eta }\mathscr {D}_0(0)[\hat{\eta }][f](x)&=-{\text {p.v.}}\int _{\mathbb {T}}\frac{\big (\hat{\eta }(y)-\hat{\eta }(x)\big )\cos (x-y)}{1-\cos (x-y)}f(y){\text {d}} y-\int _{\mathbb {T}}\big (\hat{\eta }(x)+\hat{\eta }(y)\big )f(y){\text {d}} y\\&=\frac{1}{2}{\text {p.v.}}\int _{\mathbb {T}}\frac{\hat{\eta }(x)-\hat{\eta }(y)}{\sin ^2\left( \frac{x-y}{2}\right) }f(y){\text {d}} y-2\hat{\eta }(x)\int _{\mathbb {T}}f(y){\text {d}} y. \end{aligned} \end{aligned}$$

For later purposes, we shall study the case f=1. Using Fourier symbols representation, we see that

2.23 $$\begin{aligned} |D|=\partial _{x}\mathcal {H}=\mathcal {H}\partial _x. \end{aligned}$$

In addition, since $\cot '(x)=-\frac{1}{\sin ^2(x)}$ and $\mathcal {H}[1]=0,$ we get

2.24 $$\begin{aligned} \begin{aligned} \partial _{x}\mathcal {H}[f](x)&=\partial _{x}\mathcal {H}[f](x)-f(x)\partial _{x}\mathcal {H}[1](x)\\&={\text {p.v.}}\int _{\mathbb {T}}\partial _{x}\left[ \cot \left( \frac{x-y}{2}\right) \right] \big (f(y)-f(x)\big ){\text {d}} y\\&=\frac{1}{2}{\text {p.v.}}\int _{\mathbb {T}}\frac{f(x)-f(y)}{\sin ^2\left( \frac{x-y}{2}\right) }{\text {d}} y. \end{aligned} \end{aligned}$$

Putting together (2.22), (2.23) and (2.24) yields

2.25 $$\begin{aligned} d_{\eta }\mathscr {D}_0(0)[\hat{\eta }][1]=|D|\hat{\eta }-2\hat{\eta }. \end{aligned}$$

Combining (1.7) and (2.4) and (2.21), we get

2.26 $$\begin{aligned} d_{\eta }\mathscr {H}(0)[\hat{\eta }][f]=\hat{\eta }_x\mathscr {D}_0(0)[f]+{d_{\eta }\mathscr {H}_0(0)[\hat{\eta }][f]}=\hat{\eta }_{x}\int _{\mathbb {T}}f(y){\text {d}} y. \end{aligned}$$

As a consequence, differentiating (2.1) and using (2.6), (2.23) and (2.26) implies

2.27 $$\begin{aligned} \begin{aligned} d_{\eta ,\psi }\mathcal {F}_1(c,\sigma ,\gamma ,0,0)[\hat{\eta },\hat{\psi }]&=c\,\hat{\eta }_x+\frac{1}{2}\mathscr {H}(0)[\hat{\psi }_x]+\frac{\gamma }{2}d_{\eta }\mathscr {H}(0)[\hat{\eta }][1]\\&=c\,\hat{\eta }_x+\frac{1}{2} \mathcal {H}\partial _{x}\hat{\psi }+\frac{\gamma }{2}\hat{\eta }_x\\&=\left( c+\frac{\gamma }{2}\right) \partial _{x}\hat{\eta }+\frac{|D|}{2}\hat{\psi }. \end{aligned} \end{aligned}$$

Besides, differentiating (2.2) and making appeal to (2.4), (2.20) and (2.25), we find

2.28 $$\begin{aligned} \begin{aligned} d_{\eta ,\psi }\mathcal {F}_2(c,\sigma ,\gamma ,0,0)[\hat{\eta },\hat{\psi }]&=c\,\hat{\psi }_x+\frac{\gamma }{2}\hat{\psi }_x\mathscr {D}_0(0)[1]+\frac{\gamma ^2}{2}d_{\eta }\mathscr {D}_0(0)[\hat{\eta }][1]+\frac{\gamma }{2}\mathscr {D}_0(0)[\hat{\psi }_x]+\sigma d_{\eta }\mathscr {K}(0)[\hat{\eta }]\\&=\left( c+\frac{\gamma }{2}\right) \partial _x\hat{\psi }+(\sigma -\gamma ^2)\hat{\eta }+\frac{\gamma ^2}{2}|D|\hat{\eta }-\sigma |D|^2\hat{\eta }. \end{aligned} \end{aligned}$$

Putting together (2.27) and (2.28), we obtain the matrix representation (2.18) for the linearized operator. The projection $\Pi _0^\perp$ does not alter these formulas because every displayed linearized term has zero spatial mean.

(*ii*) Coming back to the expression (2.18), we decompose the operator as follows

$$\begin{aligned} \mathcal {L}_{c,\sigma ,\gamma }=I_{\sigma }+K_{c,\sigma ,\gamma }, \end{aligned}$$

where

$$\begin{aligned} I_{\sigma }\triangleq \begin{pmatrix} 0 &  \frac{1}{2}|D|\\ -\sigma |D|^2 &  0 \end{pmatrix},\qquad K_{c,\sigma ,\gamma }\triangleq \begin{pmatrix} \left( c+\tfrac{\gamma }{2}\right) \partial _{x} &  0\\ \sigma -\gamma ^2+\tfrac{\gamma ^2}{2}|D|&  \left( c+\tfrac{\gamma }{2}\right) \partial _{x} \end{pmatrix}. \end{aligned}$$

Clearly, $I_{\sigma }:X_{\textbf{m}}^{s}\rightarrow Y_{\textbf{m}}^{s}$ is an isomorphism. In addition, $K_{c,\sigma ,\gamma }:X_{\textbf{m}}^{s}\rightarrow Y_{\textbf{m}}^{s+\frac{1}{2}}$ is continuous. By Rellich-Kondrachov Theorem, we deduce that $K_{c,\sigma ,\gamma }:X_{\textbf{m}}^{s}\rightarrow Y_{\textbf{m}}^{s}$ is a compact operator. This proves the claim by applying [62, Cor. 5.9]. □

### Bifurcation from *c*

In this subsection, we fix σ>0 and $\gamma \in \mathbb {R}$ (some restrictions will be imposed later on) and study the bifurcation from the parameter *c*. Let us look for the values of the parameter *c* such that the matrix $M_n(c,\sigma ,\gamma ),$ introduced in (2.19), is singular. For this aim, we compute its determinant

2.29 $$\begin{aligned} \det \big (M_n(c,\sigma ,\gamma )\big )=-\left( c+\frac{\gamma }{2}\right) ^2n^2+\frac{n}{4}\left( 2\sigma n^2-\gamma ^2n+2(\gamma ^2-\sigma )\right) . \end{aligned}$$

We study the sign on [1,∞) of the polynomial function

$$\begin{aligned} n\mapsto 2\sigma n^2-\gamma ^2n+2(\gamma ^2-\sigma ). \end{aligned}$$

The associated discriminant is

$$\begin{aligned} \Delta =\gamma ^4-16\sigma \gamma ^2+16\sigma ^2=(\gamma ^2-8\sigma )^2-48\sigma ^2. \end{aligned}$$

Hence,

$${2\sigma n^2-\gamma ^2n+2(\gamma ^2-\sigma )>0}\quad \Leftrightarrow \quad (n,\sigma ,\gamma )\in \mathcal {S}\triangleq \mathcal {S}_1\cup \mathcal {S}_2,$$

where

$$\begin{aligned} \mathcal {S}_1&\triangleq \big \{(n,\sigma ,\gamma )\in \mathbb {N}^*\times (0,\infty )\times \mathbb {R}\quad {\text {s.t.}}\quad 4\sigma (2-\sqrt{3})<\gamma ^2<4\sigma (2+\sqrt{3})\big \},\\ \mathcal {S}_2&\triangleq \left\{ (n,\sigma ,\gamma )\in \mathbb {N}^*\times (0,\infty )\times \mathbb {R}\quad \begin{aligned}&{\text {s.t.}}\quad \gamma ^2\in [0,\infty )\setminus \left[ 4\sigma (2-\sqrt{3}),4\sigma (2+\sqrt{3})\right] \\&{\text {and}}\quad n\in \mathbb {R}\setminus [m_-(\sigma ,\gamma ),m_+(\sigma ,\gamma )],\\&{\text {with}}\quad m_{\pm }(\sigma ,\gamma )\triangleq \frac{\gamma ^2}{4\sigma }\pm \frac{1}{4\sigma }\sqrt{(\gamma ^2-8\sigma )^2-48\sigma ^2} \end{aligned}\right\} . \end{aligned}$$

Then, for $(n,\sigma ,\gamma )\in \mathcal {S},$ we have

2.30 $$\begin{aligned} \det \big (M_n(c,\sigma ,\gamma )\big )=0\quad \Leftrightarrow \quad c=c_n^{\pm }(\sigma ,\gamma )\triangleq -\frac{\gamma }{2}\pm \frac{1}{2}\sqrt{2\sigma n-\gamma ^2+\frac{2(\gamma ^2-\sigma )}{n}}\cdot \end{aligned}$$

#### Proposition 2.3

Let $(\textbf{m},\sigma ,\gamma )\in \mathcal {S}$ satisfying the additional condition

2.31 $$\begin{aligned} \frac{\gamma ^2-\sigma }{\sigma \textbf{m}^2}\not \in \mathbb {N}^*. \end{aligned}$$

Then, the following properties hold true.

(i) The kernel of the operator $\mathcal {L}_{c_{\textbf{m}}^{\pm }(\sigma ,\gamma ),\sigma ,\gamma }$ is one dimensional. More precisely
  $$\ker \left( \mathcal {L}_{c_{\textbf{m}}^{\pm }(\sigma ,\gamma ),\sigma ,\gamma }\right) =\texttt{span}(x_{0,\sigma ,\gamma ,\textbf{m}}^{\pm }),\qquad x_{0,\sigma ,\gamma ,\textbf{m}}^{\pm }(x)\triangleq \begin{pmatrix} \cos (\textbf{m}x)\\ \pm \sqrt{2\sigma \textbf{m}-\gamma ^2+\frac{2(\gamma ^2-\sigma )}{\textbf{m}}}\sin (\textbf{m}x) \end{pmatrix}.$$
(ii) The operator $\mathcal {L}_{c_{\textbf{m}}^{\pm }(\sigma ,\gamma ),\sigma ,\gamma }:X_{\textbf{m}}^{s}\longrightarrow Y_{\textbf{m}}^{s}$ is Fredholm with zero index.
(iii) The transversality condition holds, namely
  2.32 $$\begin{aligned} \partial _c\mathcal {L}_{c,\sigma ,\gamma }|_{c=c_{\textbf{m}}^{\pm }(\sigma ,\gamma )}\left[ x_{0,\sigma ,\gamma ,\textbf{m}}^{\pm }\right] \not \in R\left( \mathcal {L}_{c_{\textbf{m}}^{\pm }(\sigma ,\gamma ),\sigma ,\gamma }\right) . \end{aligned}$$

#### Proof

(*i*) Let us study the spectral collisions of the $\textbf{m}$-fold spectrum. More precisely, we shall solve

2.33 $$\begin{aligned} c_{\textbf{m}}^{\kappa _1}(\sigma ,\gamma )=c_{k\textbf{m}}^{\kappa _2}(\sigma ,\gamma ),\qquad (\kappa _1,\kappa _2)\in \{-,+\}^2,\qquad k\in \mathbb {N}^*. \end{aligned}$$

First observe that there can exist values of $k\in \mathbb {N}\setminus \{0,1\}$ such that $(k\textbf{m},\sigma ,\gamma )\not \in \mathcal {S}$. In this case, $c_{k\textbf{m}}^{\kappa _2}(\sigma ,\gamma )$ is either equal to $-\frac{\gamma }{2}$ or an element of $\mathbb {C}\setminus \mathbb {R}$. In both cases the equation (2.33) is not satisfied. So we can restrict the discussion to the case where $(k\textbf{m},\sigma ,\gamma )\in \mathcal {S}.$ Coming back to the expression of $c_n^{\pm }(\sigma ,\gamma )$ in (2.30), the equation (2.33) implies

$$\begin{aligned} \sigma \textbf{m}+\frac{\gamma ^2-\sigma }{\textbf{m}}=\sigma k\textbf{m}+\frac{\gamma ^2-\sigma }{k\textbf{m}} \end{aligned}$$

or equivalently

$$\begin{aligned} \sigma \textbf{m}^2(k-1)\left( k-\frac{\gamma ^2-\sigma }{\sigma \textbf{m}^2}\right) =\sigma \textbf{m}^2k^2-\left( \sigma \textbf{m}^2+\gamma ^2-\sigma \right) k+\gamma ^2-\sigma =0. \end{aligned}$$

Therefore, there are two solutions

$$\begin{aligned} k_1=1,\qquad k_2=\frac{\gamma ^2-\sigma }{\sigma \textbf{m}^2}\cdot \end{aligned}$$

Since $c_{\textbf{m}}^{-}(\sigma ,\gamma )\ne c_{\textbf{m}}^{+}(\sigma ,\gamma ),$ the case $k=k_1=1$ corresponds to the trivial solution. Thanks to the condition (2.31), we deduce that the equation (2.33) has no non-trivial solution. As a consequence

$$\begin{aligned} \det \Big (M_{\textbf{m}}\big (c_{\textbf{m}}^{\pm }(\sigma ,\gamma ),\sigma ,\gamma \big )\Big )=0 \end{aligned}$$

and

$$\begin{aligned} \forall n\in \mathbb {N}{\setminus }\{0,1\},\quad \det \Big (M_{n\textbf{m}}\big (c_{\textbf{m}}^{\pm }(\sigma ,\gamma ),\sigma ,\gamma \big )\Big )\ne 0. \end{aligned}$$

This implies the one dimensional kernel property for the linearized operator and the generator is obtain by remarking that

$$\begin{pmatrix} 1\\ \pm \sqrt{2\sigma \textbf{m}-\gamma ^2+\frac{2(\gamma ^2-\sigma )}{\textbf{m}}} \end{pmatrix}\in \ker \Big (M_{\textbf{m}}\big (c_{\textbf{m}}^{\pm }(\sigma ,\gamma ),\sigma ,\gamma \big )\Big ).$$

(*ii*) Follows from Proposition 2.2-(*ii*).

(*iii*) We introduce on $Y_{\textbf{m}}^s$ the scalar product $\langle \cdot ,\cdot \rangle$ defined as follows: for (*f*, *g*) and $(\widetilde{f},\widetilde{g})$ in $Y_{\textbf{m}}^s$ admitting the Fourier representations

$$\begin{aligned} f(x)=\sum _{n=1}^{\infty }a_n\sin (n\textbf{m}x),\qquad \widetilde{f}(x)=\sum _{n=1}^{\infty }\widetilde{a}_n\sin (n\textbf{m}x) \end{aligned}$$

and

$$\begin{aligned} g(x)=\sum _{n=1}^{\infty }b_n\cos (n\textbf{m}x),\qquad \widetilde{g}(x)=\sum _{n=1}^{\infty }\widetilde{b}_n\cos (n\textbf{m}x), \end{aligned}$$

with $a_n,\widetilde{a}_n,b_n,\widetilde{b}_n\in \mathbb {R}$, their scalar product is given by

$$\begin{aligned} \langle (f,g),(\widetilde{f},\widetilde{g})\rangle \triangleq \sum _{n=1}^{\infty }a_n\widetilde{a}_n+b_n\widetilde{b}_n. \end{aligned}$$

Define

$$y_{0,\sigma ,\gamma ,\textbf{m}}^{\pm }(x)\triangleq \begin{pmatrix} \mp \sqrt{2\sigma \textbf{m}-\gamma ^2+\frac{2(\gamma ^2-\sigma )}{\textbf{m}}}\sin (\textbf{m}x)\\ \cos (\textbf{m}x) \end{pmatrix}.$$

Let us prove that

2.34 $$\begin{aligned} R\left( \mathcal {L}_{c_{\textbf{m}}^{\pm }(\sigma ,\gamma ),\sigma ,\gamma }\right) =\texttt{span}\left( y_{0,\sigma ,\gamma ,\textbf{m}}^{\pm }\right) ^{\perp _{\langle \cdot ,\cdot \rangle }}. \end{aligned}$$

Take an element $y\in R\left( \mathcal {L}_{c_{\textbf{m}}^{\pm }(\sigma ,\gamma ),\sigma ,\gamma }\right) .$ By construction,

$$y(x)=\sum _{n=1}^{\infty }\begin{pmatrix} \sin (n\textbf{m}x) &  0\\ 0 &  \cos (n\textbf{m}x) \end{pmatrix}M_{n\textbf{m}}(c,\sigma ,\gamma )\begin{pmatrix} a_n\\ b_n \end{pmatrix}.$$

Then,

$$\begin{aligned} \left\langle y,y_0^{\pm }\right\rangle&=\left\langle M_{\textbf{m}}\left( c_{\textbf{m}}^{\pm }(\sigma ,\gamma ),\sigma ,\gamma \right) \begin{pmatrix} a_n\\ b_n \end{pmatrix},\begin{pmatrix} \mp \sqrt{2\sigma \textbf{m}-\gamma ^2+\frac{2(\gamma ^2-\sigma )}{\textbf{m}}}\\ 1 \end{pmatrix}\right\rangle _{\mathbb {R}^2}\\&=\left\langle \begin{pmatrix} a_n\\ b_n \end{pmatrix},M_{\textbf{m}}^{\top }\left( c_{\textbf{m}}^{\pm }(\sigma ,\gamma ),\sigma ,\gamma \right) \begin{pmatrix} \mp \sqrt{2\sigma \textbf{m}-\gamma ^2+\frac{2(\gamma ^2-\sigma )}{\textbf{m}}}\\ 1 \end{pmatrix}\right\rangle _{\mathbb {R}^2}\\&=0. \end{aligned}$$

The last identity is obtained because by construction

$$\begin{pmatrix} \mp \sqrt{2\sigma \textbf{m}-\gamma ^2+\frac{2(\gamma ^2-\sigma )}{\textbf{m}}}\\ 1 \end{pmatrix}\in \ker \Big (M_{\textbf{m}}^{\top }\big (c_{\textbf{m}}^{\pm }(\sigma ,\gamma ),\sigma ,\gamma \big )\Big ).$$

Recall that the notation $M^{\top }$ denotes the transpose of the matrix *M*. This proves that

2.35 $$\begin{aligned} R\left( \mathcal {L}_{c_{\textbf{m}}^{\pm }(\sigma ,\gamma ),\sigma ,\gamma }\right) \subset \texttt{span}\left( y_{0,\sigma ,\gamma ,\textbf{m}}^{\pm }\right) ^{\perp _{\langle \cdot ,\cdot \rangle }}. \end{aligned}$$

Now, since the space $\texttt{span}\left( y_{0,\sigma ,\gamma ,\textbf{m}}^{\pm }\right)$ is of finite dimension, then we can apply the orthogonal supplementary Theorem in the inner product space $(Y_{\textbf{m}}^{s},\langle \cdot ,\cdot \rangle )$ to get

$$\begin{aligned} Y_{\textbf{m}}^{s}=\texttt{span}\left( y_{0,\sigma ,\gamma ,\textbf{m}}^{\pm }\right) \overset{\perp }{\oplus }\texttt{span}\left( y_{0,\sigma ,\gamma ,\textbf{m}}^{\pm }\right) ^{\perp _{\langle \cdot ,\cdot \rangle }}. \end{aligned}$$

This proves that $\texttt{span}\left( y_{0,\sigma ,\gamma ,\textbf{m}}^{\pm }\right) ^{\perp _{\langle \cdot ,\cdot \rangle }}$ is of codimension one in $Y_{\textbf{m}}^{s}.$ Besides, the points (*i*) and (*ii*) give that $R\left( \mathcal {L}_{c_{\textbf{m}}^{\pm }(\sigma ,\gamma ),\sigma ,\gamma }\right)$ is also of codimension one in $Y_{\textbf{m}}^{s}.$ Together with the inclusion (2.35), we conclude (2.34). With this in hand, we can now check the transversality condition. Notice that, from (2.18), we get

$$\partial _{c}\mathcal {L}_{c,\sigma ,\gamma }|_{c=c_{\textbf{m}}^{\pm }(\sigma ,\gamma )}=\begin{pmatrix} \partial _{x} &  0\\ 0 &  \partial _{x} \end{pmatrix}.$$

Thus, a straightforward computation together with the fact that $(\textbf{m},\sigma ,\gamma )\in \mathcal {S}$ give

$$\begin{aligned} \left\langle \partial _c\mathcal {L}_{c,\sigma ,\gamma }|_{c=c_{\textbf{m}}^{\pm }(\sigma ,\gamma )}\left[ x_{0,\sigma ,\gamma ,\textbf{m}}^{\pm }\right] ,y_{0,\sigma ,\gamma ,\textbf{m}}^{\pm }\right\rangle =\pm 2\textbf{m}\sqrt{2\sigma \textbf{m}-\gamma ^2+\frac{2(\gamma ^2-\sigma )}{\textbf{m}}}\ne 0. \end{aligned}$$

According to (2.34), this proves the transversality condition (2.32) and achieves the proof of Proposition 2.3. □

*Proof of Theorem* 1.1-*(i)*

We apply Theorem A.1 together with Lemma 2.1 and Propositions 2.1 and 2.3. □

### Bifurcation from σ

In this subsection, we fix $(c,\gamma )\in \mathbb {R}^2$ (some restrictions will be imposed later on) and study the bifurcation from the parameter σ. According to (2.29), we have

2.36 $$\begin{aligned} \det \big (M_1(c,\sigma ,\gamma )\big )=-c(c+\gamma ) \end{aligned}$$

is independent of σ and, for any n⩾2,

2.37 $$\begin{aligned} \det \big (M_n(c,\sigma ,\gamma )\big )=0\quad \Leftrightarrow \quad \sigma =\sigma _n(c,\gamma )\triangleq \frac{\left[ \left( 2c+\gamma \right) ^2+\gamma ^2\right] n-2\gamma ^2}{2(n^2-1)}\cdot \end{aligned}$$

In the sequel, we denote

$$\alpha (c,\gamma )\triangleq \left( 2c+\gamma \right) ^2+\gamma ^2,\qquad \beta (\gamma )\triangleq 2\gamma ^2.$$

The condition σ>0 requires

$$n\alpha (c,\gamma )-\beta (\gamma )>0,\qquad {\text {i.e.}}\qquad n>N(c,\gamma )\triangleq \frac{2\gamma ^2}{\left( 2c+\gamma \right) ^2+\gamma ^2}\cdot $$

#### Proposition 2.4

Let $(c,\gamma )\in \mathbb {R}^2$ and $\textbf{m}\in \mathbb {N}\setminus \{0,1\}$ with $\textbf{m}>N(c,\gamma ).$ Assume in addition that

2.38 $$\begin{aligned} {\frac{(2\textbf{m}-1)\gamma ^2-(2c+\gamma )^2}{\textbf{m}\big (\textbf{m}(2c+\gamma )^2+(\textbf{m}-2)\gamma ^2\big )}\not \in \mathbb {N}^*.} \end{aligned}$$

Then, the following properties hold true.

(i) The kernel of the operator $\mathcal {L}_{c,\sigma _{\textbf{m}}(c,\gamma ),\gamma }$ is one dimensional. More precisely,
  $$\ker \left( \mathcal {L}_{c,\sigma _{\textbf{m}}(c,\gamma ),\gamma }\right) =\texttt{span}(x_{0,c,\gamma ,\textbf{m}}),\qquad x_{0,c,\gamma ,\textbf{m}}(x)\triangleq \begin{pmatrix} \cos (\textbf{m}x)\\ (2c+\gamma )\sin (\textbf{m}x) \end{pmatrix}.$$
(ii) The operator $\mathcal {L}_{c,\sigma _{\textbf{m}}(c,\gamma ),\gamma }:X_{\textbf{m}}^{s}\longrightarrow Y_{\textbf{m}}^{s}$ is Fredholm with zero index.
(iii) The transversality condition holds, namely
  2.39 $$\begin{aligned} \partial _\sigma \mathcal {L}_{c,\sigma ,\gamma }|_{\sigma =\sigma _{\textbf{m}}(c,\gamma )}\left[ x_{0,c,\gamma ,\textbf{m}}\right] \not \in R\left( \mathcal {L}_{c,\sigma _{\textbf{m}}(c,\gamma ),\gamma }\right) . \end{aligned}$$

#### Proof

(*i*) Let us study the spectral collisions. We need to solve

2.40 $$\begin{aligned} \sigma _{\textbf{m}}(c,\gamma )=\sigma _{k\textbf{m}}(c,\gamma ),\qquad k\in \mathbb {N}^*. \end{aligned}$$

In what follows, we simply denote $\alpha \triangleq \alpha (c,\gamma )$ and $\beta \triangleq \beta (\gamma ).$ Coming back to the expression (2.37), the previous equation is equivalent to

$$\begin{aligned} (\alpha \textbf{m}-\beta )(k^2\textbf{m}^2-1)=(\alpha k\textbf{m}-\beta )(\textbf{m}^2-1), \end{aligned}$$

or again

$$\begin{aligned} (\alpha \textbf{m}-\beta )\textbf{m}^2(k-1)\left( k-\frac{\beta \textbf{m}-\alpha }{\textbf{m}(\alpha \textbf{m}-\beta )}\right) =(\alpha \textbf{m}-\beta )\textbf{m}^2k^2-\alpha \textbf{m}(\textbf{m}^2-1)k+\textbf{m}(\beta \textbf{m}-\alpha )=0. \end{aligned}$$

Therefore, the equation (2.40) admits two solutions

$$\begin{aligned} k_1=1\ {\text {(trivial solution)}},\qquad k_2=\frac{\beta \textbf{m}-\alpha }{\textbf{m}(\alpha \textbf{m}-\beta )}=\frac{(2\textbf{m}-1)\gamma ^2-(2c+\gamma )^2}{\textbf{m}\big (\textbf{m}(2c+\gamma )^2+(\textbf{m}-2)\gamma ^2\big )}\cdot \end{aligned}$$

The condition (2.38) implies that the equation (2.40) has no non-trivial solution. Then, we can conclude as in the proof of Proposition 2.3-(*i*).

(*ii*) Follows from Proposition 2.2-(*ii*).

(*iii*) Proceeding as in the proof of Proposition 2.3-(*iii*), we get

$$R\left( \mathcal {L}_{c,{\sigma _{\textbf{m}}(c,\gamma )},\gamma }\right) =\texttt{span}(y_{0,c,\gamma ,\textbf{m}})^{\perp _{\langle \cdot ,\cdot \rangle }},\qquad y_{0,c,\gamma ,\textbf{m}}(x)\triangleq \begin{pmatrix} -(2c+\gamma )\sin (\textbf{m}x)\\ \cos (\textbf{m}x) \end{pmatrix}.$$

From the expression (2.18), we see that

$$\partial _{\sigma }\mathcal {L}_{c,\sigma ,\gamma }|_{\sigma =\sigma _{\textbf{m}}(c,\gamma )}=\begin{pmatrix} 0 &  0\\ {\text {Id}}-|D|^2 &  0 \end{pmatrix}.$$

Therefore, since $$\textbf{m}\ne 1,$$

$$\begin{aligned} \left\langle \partial _{\sigma }\mathcal {L}_{c,\sigma ,\gamma }|_{\sigma =\sigma _{\textbf{m}}(c,\gamma )}[x_{0,c,\gamma ,\textbf{m}}],y_{0,c,\gamma ,\textbf{m}}\right\rangle =1-\textbf{m}^2\ne 0. \end{aligned}$$

This concludes (2.39) and the proof of Proposition 2.4. □

*Proof of Theorem* 1.1-*(ii)*

We apply Theorem A.1 together with Lemma 2.1 and Propositions 2.1 and 2.4. □

### Direct Verification for the Stationary Branches

In this subsection, we fix c=0 and σ>0. Then, we study the bifurcation from the parameter γ. According to (2.36), we get

$$\begin{aligned} \det \big (M_1(0,\sigma ,\gamma )\big )=0, \end{aligned}$$

and in view of (2.29), for any n⩾2, we have

$$\det \big (M_n(0,\sigma ,\gamma )\big )=0\quad \Leftrightarrow \quad \gamma =\gamma _n^{\pm }(\sigma )\triangleq \pm \sqrt{\sigma (n+1)}.$$

#### Proposition 2.5

Let σ>0 and $\textbf{m}\in \mathbb {N}\setminus \{0,1\}.$ Then, the following properties hold true.

(i) The kernel of the operator $\mathcal {L}_{0,\sigma ,\gamma _{\textbf{m}}^{\pm }(\sigma )}$ is one dimensional. More precisely,
  $$\ker \left( \mathcal {L}_{0,\sigma ,\gamma _{\textbf{m}}^{\pm }(\sigma )}\right) =\texttt{span}(x_{0,\sigma ,\textbf{m}}^{\pm }),\qquad x_{0,\sigma ,\textbf{m}}^{\pm }(x)\triangleq \begin{pmatrix} \cos (\textbf{m}x)\\ \gamma _{\textbf{m}}^{\pm }(\sigma )\sin (\textbf{m}x) \end{pmatrix}.$$
(ii) The operator $\mathcal {L}_{0,\sigma ,\gamma _{\textbf{m}}^{\pm }(\sigma )}:X_{\textbf{m}}^{s}\longrightarrow Y_{\textbf{m}}^{s}$ is Fredholm with zero index.
(iii) The transversality condition holds, namely
  2.41 $$\begin{aligned} \partial _{\gamma }\mathcal {L}_{0,\sigma ,\gamma }|_{\gamma =\gamma _{\textbf{m}}^{\pm }(\sigma )}\left[ x_{0,\sigma ,\textbf{m}}^{\pm }\right] \not \in R\left( \mathcal {L}_{0,\sigma ,\gamma ^{\pm }_{\textbf{m}}(\sigma )}\right) . \end{aligned}$$

#### Proof

(*i*) Observe that $\gamma _{\textbf{m}}^-(\sigma )=-\gamma _{\textbf{m}}^+(\sigma )\ne 0$ and that the sequence $\big (\gamma _{n}^{+}(\sigma )\big )_{n\geqslant 2}$ is strictly increasing. This immediately prevents spectral collisions and allows to conclude similarly to Proposition 2.3-(*i*).

(*ii*) Follows from Proposition 2.2-(ii).

(*iii*) Proceeding as in the proof of Proposition 2.3-(*iii*), we get

$$R\left( \mathcal {L}_{{0},\sigma ,{\gamma _{\textbf{m}}^{\pm }(\sigma )}}\right) =\texttt{span}(y_{0,\sigma ,\textbf{m}}^{\pm })^{\perp _{\langle \cdot ,\cdot \rangle }},\qquad y_{0,\sigma ,\textbf{m}}^{\pm }(x)\triangleq \begin{pmatrix} -\gamma _{\textbf{m}}^{\pm }(\sigma )\sin (\textbf{m}x)\\ \cos (\textbf{m}x) \end{pmatrix}.$$

From (2.18), we have

$$\partial _{\gamma }\mathcal {L}_{0,\sigma ,\gamma }|_{\gamma =\gamma _{\textbf{m}}^{\pm }(\sigma )}=\begin{pmatrix} \frac{1}{2}\partial _{x} &  0\\ \gamma _{\textbf{m}}^{\pm }(\sigma )(|D|-2) &  \frac{1}{2}\partial _{x} \end{pmatrix}.$$

Therefore, since $\textbf{m}\ne 1$ and $\gamma _{\textbf{m}}^{\pm }(\sigma )\ne 0,$

$$\begin{aligned} \left\langle \partial _{\gamma }\mathcal {L}_{{0},\sigma ,\gamma }|_{\gamma =\gamma _{\textbf{m}}^{\pm }(\sigma )}[x_{0,\sigma ,\textbf{m}}^{\pm }],y_{0,\sigma ,\textbf{m}}^{\pm }\right\rangle =2\gamma _{\textbf{m}}^{\pm }(\sigma )\left( \textbf{m}-1\right) \ne 0. \end{aligned}$$

This implies (2.41) and achieves the proof of Proposition 2.5. □

#### Direct proof of Corollary 1.1

We apply Theorem A.1 together with Lemma 2.1 and Propositions 2.1 and 2.5. This gives an alternative direct verification of the stationary branches already deduced from scaling. □

## Data Availability

No data has been used in this work.

## Appendix

### Derivation of (1.4) from (1.1)

From (1.3) it is immediate that we can recast (1.1) as an evolutionary equation on the interface only, since the bulk quantities $u^\pm$ and $p^\pm$ can be recovered from the interface evolution of ω and *z*. Such derivation is quite well-known in the literature (cf. [63, 64]) but we perform here detailed computations for the sake of clarity.

Given two functions $f^{\pm }:\Omega ^{\pm }(t)\rightarrow \mathbb {R}$ we define

$$\begin{aligned} {\llbracket f^{\pm } \rrbracket \triangleq f^+-f^-.} \end{aligned}$$

Let us now define the trace of the velocity $u^\pm$ (cf. (1.3)) as

A.1 $$\begin{aligned} v^{\pm }(t,x)\triangleq \left. u^{\pm }\right| _{\Gamma (t)}(t,x)=u^{\pm }\big (t,z(t,x)\big ),\qquad (t,x)\in (0,T)\times \mathbb {T}. \end{aligned}$$

From (1.3) we can compute $v^\pm$ defined in (A.1) as

$$\begin{aligned} {v^{\pm }\left( t, x \right) = \lim _{\epsilon \searrow 0} u^{\pm }\left( t, z\left( t, x \right) \mp \epsilon z_x^\perp \left( t, x \right) \right) } \end{aligned}$$

since $z\left( t, x \right) \mp \epsilon z_x^\perp \left( t, x \right) \in \Omega ^{\pm }\left( t \right)$ for any $x\in \mathbb {T}$, so that the trace of the velocity flow $v^{\pm }$ can be recasted in terms of *z* and ω via the *Birkhoff-Rott integral operator*

A.2 $$\begin{aligned} v^{\pm }={\text {BR}}(z)\omega \pm \frac{\omega }{2}\frac{z_x}{\left| z_x\right| ^2},\qquad {\text {BR}}(z) \omega \left( t,x \right) \triangleq {\text {p.v.}}\int _\mathbb {T}\frac{\left( z\left( t,x \right) -z\left( t,y \right) \right) ^\perp }{\left| z\left( t,x \right) -z\left( t,y \right) \right| ^2} \omega \left( t, y \right) {\text {d}}y. \end{aligned}$$

The integral uses the normalized measure on $\mathbb {T}$ fixed after (1.3); hence no additional factor 1/(2π) occurs in (A.2).

From (A.2) we can deduce hence a relation between the vorticity strength and the trace of the velocity valid in $\left( 0, T \right) \times \mathbb {T}$ (cf. (A.1))

A.3 $$\begin{aligned} \omega = \llbracket v^{\pm } \rrbracket \cdot z_x. \end{aligned}$$

#### Remark A.1

Notice that from (A.2) it is immediate that the normal (to $\Gamma \left( t \right)$) component of the velocity flow is continuous through the interface.

The relation (A.3) allows us to express ω in terms of the trace of the velocity flow, hence, to derive the evolution equation for ω by taking the tangential (to $\Gamma \left( t \right)$) trace of the first equation of (1.1) onto $\Gamma \left( t \right)$. In the present model there is no gravity term, and the tangential trace of the Euler equation is

A.4 $$\begin{aligned} {\left( v^{\pm } \cdot z_x \right) _t - \left( v^\pm \cdot z_t \right) _x + \frac{1}{2}\left( \left| v^{\pm }\right| ^2 \right) _x + \left( \left. p^\pm \right| _{\Gamma \left( t \right) } \right) _x = 0.} \end{aligned}$$

The term $(z_2)_x$ in the previous version belongs to the formulation with vertical gravity and was inadvertently retained from [64, Eq. (2.2)]; removing it restores rotational invariance. Using (A.4) we can hence compute the evolution equation for ω defined as in (A.3) which is, using the pressure jump in the third equation of (1.1),

A.5 $$\begin{aligned} \omega _t-\left( \llbracket v^\pm \rrbracket \cdot z_t \right) _x+\frac{1}{2}\left( \llbracket \left| v^\pm \right| ^2 \rrbracket \right) _x+\sigma \big (\mathcal {K}(z)\big )_x=0, \end{aligned}$$

while we use (A.2) in order to obtain the identity

$$\begin{aligned} {v^+=v^- + \omega \ \frac{z_x}{\left| z_x\right| ^2},} \end{aligned}$$

from which we derive

A.6 $$\begin{aligned} {v^+\cdot z_x = v^-\cdot z_x + \omega },  &   {v^+\cdot z_t = v^-\cdot z_t + \omega \frac{z_t\cdot z_x}{\left| z_x\right| ^2}},  &   {\left| v^+\right| ^2 = \left| v^-\right| ^2 + \frac{\omega ^2}{\left| z_x\right| ^2} + 2\omega \ \frac{v^-\cdot z_x}{\left| z_x\right| ^2}}\cdot \end{aligned}$$

The relations in (A.6) and (A.2) give us that

A.7 $$\begin{aligned} -\left( \llbracket v^\pm \rrbracket \cdot z_t \right) _x+\frac{1}{2}\left( \llbracket \left| v^\pm \right| ^2 \rrbracket \right) _x={\frac{1}{2}\left( \frac{\omega ^2}{\left| z_x\right| ^2} \right) _x-\left( \omega \ \frac{\left( z_t-v^- \right) \cdot z_x}{\left| z_x\right| ^2} \right) _x} =-\left( \omega \ \frac{\left( z_t-{\text {BR}}(z)\omega \right) \cdot z_x}{\left| z_x\right| ^2} \right) _x. \end{aligned}$$

The system (1.1) can thus be recasted as an evolutionary equation on the interface only via the unknowns $\left( \omega , z \right)$, plugging (A.7) in (A.5), thus obtaining (1.4).

### Derivation of (1.6) from (1.4)

Here we explain how to get the system (1.6) from (1.4). Let us consider a parametrization of Γ(t) in the form

$$z(t,x)=R(t,x)e^{\textrm{i} x},\qquad R(t,x)\triangleq \sqrt{1+2\eta (t,x)}.$$

Straightforward calculations show that

A.8 $$\begin{aligned} \begin{aligned} z_{t}(t,x)&=\frac{\eta _t(t,x)}{R(t,x)}e^{\textrm{i} x},\\ z_{x}(t,x)&=\left( \frac{\eta _x(t,x)}{R(t,x)}+\textrm{i} R(t,x)\right) e^{\textrm{i} x},\\ z_{x}^{\perp }(t,x)&=\left( \textrm{i} \frac{\eta _x(t,x)}{R(t,x)}-R(t,x)\right) e^{\textrm{i} x},\\ z_{xx}(t,x)&=\left( \frac{\eta _{xx}(t,x)}{R(t,x)}-\frac{\eta _{x}^2(t,x)}{R^3(t,x)}+2\textrm{i} \frac{\eta _{x}(t,x)}{R(t,x)}-R(t,x)\right) e^{\textrm{i} x}. \end{aligned} \end{aligned}$$

As a consequence,

A.9 $$\begin{aligned} \begin{aligned} z_t\cdot z_{x}^{\perp }&=-\eta _{t},\\ z_{t}\cdot z_{x}&=\frac{\eta _{t}\eta _x}{R^2},\\ z_{x}^{\perp }\cdot z_{xx}&=3\left( \frac{\eta _x}{R}\right) ^2-\eta _{xx}+R^2,\\ |z_x|^2&=R^2+\left( \frac{\eta _{x}}{R}\right) ^2. \end{aligned} \end{aligned}$$

The last two identities combined with (1.10) give immediately

A.10 $$\begin{aligned} \mathcal {K}(z)=\frac{\eta _{xx}-2\left( \frac{\eta _{x}}{R}\right) ^2}{\left( R^2+\left( \frac{\eta _{x}}{R}\right) ^2\right) ^{\frac{3}{2}}}-\left( R^2+\left( \frac{\eta _{x}}{R}\right) ^2\right) ^{-\frac{1}{2}}=\mathscr {K}(\eta ). \end{aligned}$$

In addition,

A.11 $$\begin{aligned} \begin{aligned} \big (z(x)-z(y)\big )\cdot z_{x}(x)&=\eta _{x}(x)\left( 1-\frac{R(y)}{R(x)}\cos (x-y)\right) +R(x)R(y)\sin (x-y),\\ |z(x)-z(y)|^2&=2\big (1+\eta (x)+\eta (y)-R(x)R(y)\cos (x-y)\big ). \end{aligned} \end{aligned}$$

Using (A.11) and the notation (1.7), we deduce that

$$\begin{aligned} {\text {BR}}(z)\omega \cdot z_{x}^{\perp }(x)&={\text {p.v.}}\int _{\mathbb {T}}\frac{\big (z(x)-z(y)\big )^{\perp }\cdot z_{x}^{\perp }(x)}{|z(x)-z(y)|^2}\omega (y){\text {d}} y\\&={\text {p.v.}}\int _{\mathbb {T}}\frac{\big (z(x)-z(y)\big )\cdot z_{x}(x)}{|z(x)-z(y)|^2}\omega (y){\text {d}} y\\&=\tfrac{1}{2}\mathscr {H}(\eta )[\omega ](x). \end{aligned}$$

Together with (A.9), the first equation in (1.4) becomes

A.12 $$\begin{aligned} \eta _{t}=-\tfrac{1}{2}\mathscr {H}(\eta )[\omega ]. \end{aligned}$$

Combining (1.4), (A.8) and (A.9), we find

$$\begin{aligned} z_{t}\cdot z_{x}=-\frac{\eta _{x}}{R^2}{\text {BR}}(z)\omega \cdot z_{x}^{\perp }. \end{aligned}$$

Thus,

$$\begin{aligned} \big (z_{t}-{\text {BR}}(z)\omega \big )\cdot z_{x}&=-{\text {BR}}(z)\omega \cdot \left( \frac{\eta _{x}}{R^2}z_{x}^{\perp }+z_{x}\right) \\&=-|z_{x}|^2{\text {BR}}(z)\omega \cdot \left( \frac{\textrm{i} e^{\textrm{i} x}}{R}\right) . \end{aligned}$$

But

$$\begin{aligned} \big (z(x)-z(y)\big )^{\perp }\cdot \left( \frac{\textrm{i} e^{\textrm{i} x}}{R}\right) =1-\frac{R(y)}{R(x)}\cos (x-y). \end{aligned}$$

The last computations with (A.11) and (1.8) give

$$\begin{aligned} \omega \frac{\big (z_{t}-{\text {BR}}(z)\omega \big )\cdot z_{x}}{|z_x|^2}(x)&=-\omega (x){\text {p.v.}}\int _{\mathbb {T}}\frac{\big (z(x)-z(y)\big )^{\perp }\cdot \left( \tfrac{\textrm{i} e^{\textrm{i} x}}{R}\right) }{|z(x)-z(y)|^2}\omega (y){\text {d}} y\\&=-\tfrac{1}{2}\omega (x)\mathscr {D}_{0}(\eta )[\omega ](x). \end{aligned}$$

This together with (1.4) and (A.10) imply

$$\begin{aligned} \omega _{t}=\Big (-\tfrac{1}{2}\omega \mathscr {D}_{0}(\eta )[\omega ]-\sigma \mathscr {K}(\eta )\Big )_x. \end{aligned}$$

### Crandall-Rabinowitz Theorem

We state here the local bifurcation result obtained in [65] and used in this study to construct our solutions.

#### Theorem A.1

(Crandall–Rabinowitz)

Let *X* and *Y* be Banach spaces, let $(p_0,u_0)\in \mathbb {R}\times X$, and let *U* be an open neighborhood of $(p_0,u_0)$ in $\mathbb {R}\times X$. Consider a map $F\in C^2(U;Y)$ such that

(i) $F(p,u_0)=0$ for every *p* such that $(p,u_0)\in U$;
(ii) $d_uF(p_0,u_0)$ is Fredholm of index zero and, for some $x_0\in X\setminus \{0\}$,
  $$\begin{aligned} \ker \big (d_uF(p_0,u_0)\big )=\texttt{span}(x_0); \end{aligned}$$
(ii) the transversality condition
  $$\begin{aligned} \partial _pd_uF(p_0,u_0)[x_0]\notin R\big (d_uF(p_0,u_0)\big ) \end{aligned}$$
  holds.

Let *Z* be a closed complement of $$\texttt{span}(x_0)$$ in *X*. Then there exist ϵ>0, an open neighborhood $U^{\prime }\subset U$ of $$(p_0,u_0)$$, and $$C^1$$ maps

$$\begin{aligned} p:(-\epsilon ,\epsilon )\longrightarrow \mathbb {R},\qquad z:(-\epsilon ,\epsilon )\longrightarrow Z, \end{aligned}$$

with $$p(0)=p_0$$ and z(0)=0, such that the zero set of *F* in $U^{\prime }$ is precisely

$$\begin{aligned} \big \{(p,u_0)\in U'\big \}\cup \big \{\big (p(\texttt{s}),u_0+\texttt{s}x_0+\texttt{s}z(\texttt{s})\big ):|\texttt{s}|<\epsilon \big \}. \end{aligned}$$
